# Tunisians peoples dealing with death in COVID-19 pandemic: Lived experiences of grief

**DOI:** 10.1192/j.eurpsy.2021.1749

**Published:** 2021-08-13

**Authors:** H. Ghabi, A. Aissa, S. Meddouri, U. Ouali, F. Nacef

**Affiliations:** Psychiatry A, Razi Hospital, Manouba, Tunisia

**Keywords:** Grief, Muslim tunisian family, COVID-19, lived experiences

## Abstract

**Introduction:**

As Dame Cecily Saunders said, “How people die remains in the memory of those who live on.” For Muslim people, funerals and burial procedures are crucial moments that help them come to terms with the loss of a loved one. The COVID-19 pandemic has disrupted usual experiences of grief since funerals and burials are held without the presence of family. Approaches to support grief are needed to be adapted to these particular circumstances.

**Objectives:**

Describe the lived experiences of grief of the Muslim Tunisian family for patients who died due to COVID -19.

**Methods:**

This was a qualitative study with a phenomenological approach. Data of patients who died due to COVID -19 were collected. One family member or more of each deceased was contacted. Semi directive interview was conducted to help participants to describe the lived experience.

**Results:**

30 persons participated in this study. The reactions of participants towards death were crying, being sad, and being choked. The reactions of grief were influenced by several factors. These included: the circumstances of the deceased, relationship with him, the hospitalization in an intensive care unit, doctors’ expectation, and the average length of stay in hospitals before the death. Islamic religious beliefs influenced the way family experienced grief, mainly toward the management of the dead body and the imposed funeral protocol.

**Conclusions:**

This study describes the devastating impact of COVID-19 toward lived experiences of grief of Muslim Tunisian. In light of these results, grief therapies should be adapted and evaluated in this population.

**Disclosure:**

No significant relationships.

